# Post‐surgery level of circulating DNA in stage III colon cancer patients: Impact on the reliability of minimal residual disease detection

**DOI:** 10.1002/ijc.70370

**Published:** 2026-02-11

**Authors:** Andrei Kudriavtsev, Saidi Daoud, Catalina Isabel Cofre Muñoz, Alexia Mirandola, Ekaterina Pisareva, Javier Gonzalo Ruiz, Marco Macagno, Nadia Saoudi Gonzalez, Evelyne Crapez, Marc Ychou, Ramon Salazar Soler, Elisabetta Fenocchio, Paula X. Fernandez Calotti, Thibault Mazard, Cristina Santos Vivas, Elena Elez, Federica Di Nicolantonio, Alain R. Thierry

**Affiliations:** ^1^ IRCM Montpellier Cancer Research Institute, INSERM U1194, Montpellier University Montpellier France; ^2^ Medical Oncology Department Institut Català d'Oncologia (ICO)—IDIBELL Barcelona Spain; ^3^ Medical Oncology Department VHIO Vall d'Hebron Institute of Oncology Barcelona Spain; ^4^ Istituto di Candiolo Fondazione del Piemonte per l'Oncologia—IRCCS Candiolo Torino Italy; ^5^ ICM Institut Régional du Cancer de Montpellier Montpellier France; ^6^ Faculty of Medicine and Health Sciences Universitat de Barcelona CIBERONC Barcelona Spain; ^7^ Department of Oncology University of Torino Turin Italy

**Keywords:** adjuvant therapy, biomarkers, circulating DNA, colorectal cancer, diagnostic, minimal residual disease, Neutrophil extracellular traps, post‐surgery

## Abstract

Minimal residual disease (MRD) assessment using circulating nuclear DNA (cir‐nDNA) testing has demonstrated strong prognostic value in patients with operable stage III colon cancer (CC). However, further clinical research is needed to optimize the use of adjuvant therapy in this context. Since the sensitivity of variant allele frequency (VAF)‐based MRD detection depends on total cirDNA concentration, we investigated its variation in 67 stage III CC patients over 8 weeks following surgery. A majority of patients showed significantly higher post‐surgical cir‐nDNA levels compared to their pre‐surgical baseline—71.1% during the first month and 51.4% during the second. Cir‐nDNA levels tended to considerably vary (up to 18‐fold), especially during the first 3 weeks after surgery. We also observed a strong correlation between cir‐nDNA levels and neutrophil extracellular traps (NETs) markers throughout the 2‐month post‐operative period. While previous studies have generally assumed that cir‐nDNA levels decrease significantly within 1 month post‐surgery in MRD‐negative stage III patients, our findings challenge this paradigm. NETs appear to be a substantial source of cir‐nDNA and represent a major confounding factor in interpreting total cir‐nDNA, given the high inter‐individual variability in NETs production after surgery. Our data suggest that defining a single optimal time point for MRD testing may be misleading. Instead, we propose a sampling window between fourth and sixth week post‐surgery. Additionally, our results call into question the reliance on VAF as a standalone metric for MRD detection. The most robust strategy would involve integrated monitoring of total cir‐nDNA concentration, absolute mutant DNA levels, and NETs‐associated inflammation.

## What's New?

Circulating nuclear DNA (cir‐nDNA)‐based assessment of minimal residual disease (MRD) offers powerful post‐surgery prognostic insight for stage III colon cancer (CC). Sparse knowledge of post‐surgical variations and origins in cir‐nDNA, however, has limited its clinical application. Here, the authors evaluated changes in cir‐nDNA through 8 weeks following surgery in stage III CC patients. High inter‐individual variation of total cir‐nDNA concentration was observed and linked to neutrophil extracellular trap formation, a driver of cancer progression. Although cirDNA concentrations typically were elevated during the initial 2 months following surgery, the findings raise questions about optimal post‐surgery blood collection time for MRD detection.

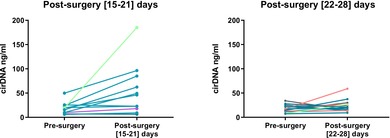

**Abbreviations**
ACT: adjuvant chemotherapy
cir‐nDNA: circulating nuclear DNA
CRC: colorectal cancer
EDTA: ethylene diamine tetraacetic acid tubes
ELISA: enzyme‐linked immunosorbent assay
HI: healthy individuals
LOD: limit of detection
LOH: loss of heterozygosity
MPO: myeloperoxidase
MRD: minimal residual disease
NE: neutrophil Elastase
NETs: neutrophil extracellular traps
VAF: variant allele frequency

## 1 INTRODUCTION

Conventional histopathological and radiological examinations show limitations in determining which patients have minimal residual disease (MRD).^1^, ^2^ Molecular MRD testing can support more individualized decisions about patient management, particularly as to the probable efficacy of treatment, in the early detection of recurrence, and with regard to prognosis.^3^, ^4^ The standard care management of patients with stage III colon cancer, or of high‐risk stage II patients, includes surgery followed by adjuvant chemotherapy (ACT).^1^, ^2^ Whereas 80% of stage II and 50% of stage III colon cancer patients are cured with surgery alone, around 15% and 30% of patients in stage II and stage III, respectively, experienced relapse despite receiving appropriate ACT.^3^ Consequently, it remains a major challenge to accurately identify those patients who ought to be administered ACT or a lower ACT dose regimen.^1^, ^2^

MRD assessment would identify patients who could benefit from adjuvant therapy, while a failure to detect MRD would spare patients who have already undergone surgery from having also to undergo adjuvant therapy, with its ensuing toxicities.^1^, ^2^, ^3^, ^4^ The detection of circulating DNA derived from malignant cells would appear to be a promising method of predicting relapse in many solid cancers.^5^, ^6^ Along with an increased exploitation of circulating nuclear DNA (cir‐nDNA)^7^ in a theragnostic strategy,^8^ MRD detection via this newly‐identified biological source has obvious advantages. Pioneering work in this field by Tie and colleagues^2^, ^9^ confirmed the benefits of stratifying patients using cir‐nDNA for detecting residual disease, particularly those with stage II and III colon cancer.

In patients with stage III colon cancer, it is current clinical practice to begin ACT between the second and eighth weeks post‐surgery. Substantial advances have been made in evaluating the performance of adjuvant therapy guided by MRD detection as determined by cir‐nDNA analysis.^1^, ^5^, ^9^ However, all such assays to date include false positive and false negative cases, and in a fraction of individuals cir‐nDNA detection cannot be evaluated,^3^, ^4^ suggesting the need for further clinical research in this area. We hypothesize that novel insights toward resolving these drawbacks could be derived from research that considers (i) the individualization of data for each patient and (ii) confounding factors with respect to the analytical signal sensitivity of MRD detection. To this end, we studied the variations and the origins of cir‐nDNA up to 8 weeks post‐surgery in each stage III colon cancer patient of the THRuST clinical study. Given that we previously showed that increases in cir‐nDNA are associated with neutrophil extracellular traps (NETs) formation in mCRC patients at diagnosis,^10^, ^11^ here we evaluated the impact of NETs formation on the variation of cir‐nDNA amounts within this post‐surgery period.

## 2 MATERIALS AND METHODS

### 2.1 Patients and cohort

In this ancillary study, we examined plasma collected in the post‐surgery period from 67 stage III colon cancer patients included as part of another study—THRuST clinical trial (015‐FPO18), which was an ERC Transcan European project (https://transcan.eu/output-results/projects/thrust.kl). The inclusion criteria selected patients aged ≥18 years old with histologically confirmed stage III colorectal adenocarcinoma. The main exclusion criteria were: active viral infection (hepatitis, HPV, HIV), previous systemic or radiation therapy for colorectal cancer, and a history of another neoplastic disease. Patients were screened and included at the IRCC (Istituto di Candiolo‐Fondazione del Piemonte per l'Oncologia, Candiolo, Italy), VHIO (Vall d'Hebron University Hospital, Vall d'Hebron Institute of Oncology, Barcelona, Spain), ICO (Catalan Institute of Oncology, L'Hospitalet de Llobregat, Barcelona, Spain) and ICM (Montpellier Cancer Institute, Montpellier, France).

**FIGURE 1:**
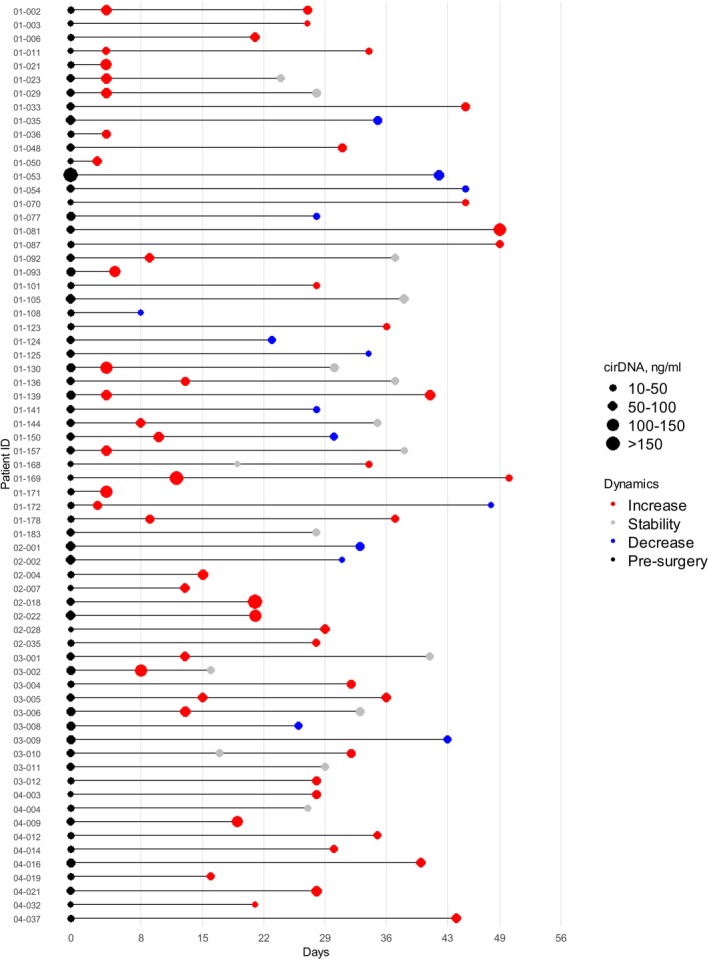
Timeline of blood sample collection. The graphs representing the diagram illustrating the timeline of blood sample collection.

The median (min–max) number of days of the first post‐operative blood sampling was 21 (3–49). A total of 67 patients were examined during the first 8 weeks post‐surgery (Figure 1), 47 were subjected to only one blood sample (median (min–max) days post‐surgery: 28 (3–49)). A total of 20 patients were subjected to two plasma samples (median (min–max) post‐surgery days of first sample: 9 (3–19) days; second sample: 35 (16–50) days). A total of 16 out of those 20 patients (80%) were subjected to a second blood sample performed between 28 and 56 days post‐surgery.

Clinical data revealed no difference in complication rates directly attributable to the surgical procedure itself in the immediate post‐surgery period. For instance, no excessive bleeding was reported. Only 5 out of the 67 examined patients experienced adverse events, but these occurred 64, 93, 545, 586, and 612 days post‐surgery. Consequently, no adverse event occurred during the 0–56 days post‐operative period.

Blood samples for this study were collected before and after surgery. The collected data was analyzed with reference to clinical observations, standard patient care management, conventional imaging methods, and conventional CRC biomarkers.

### 2.2 Blood samples collection

Each clinical center followed the same guidelines^12^ concerning blood collection in ethylene diamine tetraacetic acid tubes (EDTA), handling and storage.^12^ Each center has a specific certified and registered CC bank, which adheres to the THRuST clinical study protocol. Isolated plasma samples were stored at −80°C and handled according to cir‐nDNA preanalytic guidelines.^13^ Blood samples were collected between April 2019 and July 2023. In the course of their routine pre‐ and post‐surgical surveillance, patients were submitted to a blood sampling plan: 1 pre‐surgery, 1 or 2 post‐surgery (up to 56 days) at a visit day.

### 2.3 Plasma isolation and cir‐nDNA extraction

EDTA tubes were centrifuged at 1200*g* for 10 min at 4°C, within 4 h of collection. Plasma samples were immediately stored at −80°C and transferred on dry ice from the recruiting institutions to IRCM. Plasma was stored for a number of months (4–12 months), and centrifuged at 16,000*g* for 10 min at 4°C. An aliquot of plasma was then used to perform an enzyme‐linked immunosorbent assay (ELISA). Cir‐nDNA was extracted from 1 mL plasma (Maxwell_RSC Instrument) using the ccfDNA Plasma Kit (Promega Corporation, Madison, WI, USA) in an elution volume of 130 μL. cir‐nDNA quantification was performed in accordance with the cir‐nDNA preanalytical guidelines.^12^, ^13^ DNA extracts were stored at −20°C until use. For samples stored at −80°C^14^ for up to 3 years, to date there has been no report in the literature of any significant variation of cir‐nDNA concentration in both CC patients and healthy individuals (HI) as determined by the amplification of a 67 bp‐length wild‐type fragment of the KRAS gene.^15^ In total, 154 plasma samples from 67 patients were analyzed. Given the high specificity of our method for determining nuclear DNA concentration, we used here the term cir‐nDNA for total circulating cell free nuclear DNA, as previously reported, precluding any possible bias in the detection of mitochondrial cirDNA which might derive from other release mechanisms.^16^, ^17^

### 2.4 Quantification of cir‐nDNA

Analysis of cir‐nDNA was carried out using the multiplexed IntPlex® method which was specifically designed for quantifying cir‐nDNA.^8^ Briefly, on a CFX96 instrument using the CFX manager software (Bio‐Rad), Q‐PCR amplifications were conducted in two replicates, with each reaction having a total volume of 25 μL. Each PCR reaction comprised 12.5 μL of IQ Supermix Sybr Green (Bio‐Rad), 2.5 μL of DNase‐free water (Qiagen) or specific oligoblocker, 2.5 μL of forward and reverse primers (0.3 pmol/mL), and 5 μL of DNA extract. The thermal cycling process consisted of three repeated steps: a hot‐start activation step at 95°C for 3 min, followed by 40 denaturation–amplification cycles at 95°C for 10 s, then at 60°C for 30 s. Melting curves were studied by gradually increasing the temperature from 60 to 90°C, with a plate reading taken at every 0.2°C increment. With a genomic extract of the DiFi cell line at 1.8 ng/mL of DNA, standards curves were generated for each run, in order to maintain accuracy and consistency in the results. Each PCR run was carried out with template control and positive control for each primer set. Validations of Q‐PCR amplification were controlled by melt curve differentiation. To quantify the total cir‐nDNA concentration in both CC patients and HI, amplification of a 67 bp‐length wild‐type fragment of the KRAS gene was performed. The coefficient of variation was determined as 24% for the quantification of cir‐nDNA, when considering variation due to the extraction procedure and analysis in the same plate.^18^ The accuracy of this study's measurement of cir‐nDNA concentration is supported by two assessments. (i) The total cir‐nDNA concentration, obtained by targeting a KRAS fragment. This quality control enabled the detection and exclusion of samples which presented a loss of heterozygosity (LOH) or gene amplification, two phenomena which have been reported in CRC patients.^14^ Moreover, since KRAS amplification is an infrequent event in CRC (0.67%),^14^ its level did not impact our observations or the values described here. (ii) This method of quantifying cir‐nDNA has undergone rigorous experimental and clinical validation, demonstrating unparalleled specificity and sensitivity, to the point of permitting the detection of a single DNA fragment molecule, as determined under Poisson law distribution experiment. A healthy cir‐nDNA median value (12.6 ng/mL) was determined using a control cohort of 22 healthy individuals. To facilitate our observations, we arbitrarily defined a positivity threshold for cir‐nDNA concentration, thus enabling us to rigorously distinguish pathological or abnormal values with control values by adding the standard deviation to the median value of the healthy individuals.

### 2.5 Myeloperoxidase and neutrophil elastase assay

Myeloperoxidase (MPO) and neutrophil elastase (NE) concentrations were measured using an ELISA technique with a Duoset kit from R&D systems. This was used according to the manufacturer's standard protocol (Duoset R&D Systems, DY008, DY3174, and DY9167‐05). Both compounds synergistically contribute to Netosis, especially to DNA decondensation following neutrophil activation, and are specifically anchored to NETs filaments following NETs extracellular release. The healthy cir‐nDNA concentration median value for MPO and NE markers (12.8 and 7.6 ng/mL, respectively) was determined using a control cohort of 22 HIs (Table S4). Similar to our definition of a positivity threshold for cir‐nDNA, we arbitrarily defined a positivity threshold for MPO and NE concentration (17.0 and 10.5 ng/mL, respectively), by adding the standard deviation to the median value of HIs (Figure S1), to evaluate more stringently any discrepancies between abnormal/pathological values and healthy control values. A reproducibility test revealed a coefficient of variation of 3.14% and 5.82% for the quantification of MPO and NE, respectively, carried out in the same respective plate. A reference sample was added in triplicate in each plate to normalize the value obtained, thus addressing potential variations deriving from manipulator or plate variations. All MPO and NE measurements were carried out using the same batch of plate.

### 2.6 Statistical analysis

To investigate the associations of patients cir‐nDNA concentrations at different time points, we utilized the Mann–Whitney test. This was done subsequent to conducting the Shapiro–Wilk test, which confirmed that the sample groups did not adhere to a Gaussian distribution. Spearman correlation evaluates the strength and direction of monotonic associations between two variables. It provides a correlation coefficient to explore the relation between these continuous variables: cir‐nDNA, MPO and NE. Our guide for the interpretation of the correlation coefficient is: 0.19, no or negligible relationship; 0.20–0.29, weak but significant relationship if there is a *p*‐value <.05; 0.30–0.39; moderate but significant relationship if there is a *p*‐value <.05; 0.40–0.69; strong and significant relationship if there is a *p*‐value <.05; and, >.70, very strong and significant relationship if there is a *p*‐value <.05. All *p*‐values reported are two sided. The significance level was set at 5% (*p* <.05); *p <.05; **p <.01; ***p <.001; ****p <.0001. Statistical analysis was performed using the Graph Pad Prism 10.0.1 software.

## 3 RESULTS

In the cohort of 67 patients, 47 had a single post‐surgery sample, and 20 had two post‐surgery samples (Figure 1). For subsequent analyses comparing pre‐ and post‐surgery samples, paired values from 67 patients were utilized (Table S1). In cases where two post‐surgery samples from a patient fell within the defined timeframe, the most recent sample was prioritized for analysis. Overall, among all blood draws from this cancer cohort, there were 50 (67.6%) and 15 (20.3%) samples which showed cir‐nDNA concentrations higher and twice higher (Tables S2 and S3) than the median for HIs (Table S4), respectively (12.6 ± 7.2, SD).

Considering all available 87 post‐surgery samples, 77 (88.5%) and 49 (56.3%) plasma samples showed higher and twice higher cir‐nDNA concentrations than the median for healthy individuals, respectively (Table S3). Unexpectedly, we observed that 2 months post‐surgery 39 out of 67 (58.1%) patients showed cir‐nDNA concentrations higher than their corresponding pre‐surgery value. Notably, plasma samples from 20 (29.9%) and 14 (20.9%) patients out of 67 showed a two‐ and three‐fold increase as compared to their pre‐surgery values, respectively (Table S3A).

**FIGURE 2:**
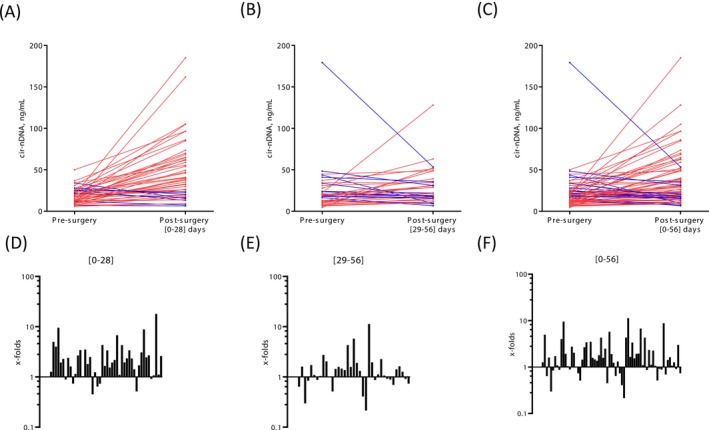
Comparative analysis of cir‐nDNA concentrations in pre‐surgery and post‐surgery. The graphs representing the comparative analysis of pre‐surgical versus post‐surgical values of cir‐nDNA for up to 28 days (A), from 29 up to 56 days (B) and up to 56 days (C). Each line represents the values for an individual patient, with red indicating an increase over time and blue indicating a decrease over time. Cir‐nDNA: Circulating nuclear DNA. Respectively, x‐fold factor indicated for the period from 0 up to 28 days (D), from 29 up to 56 days (E) and up to 56 days (F).

Considering that the time to recover from surgery can impact cir‐nDNA levels, we performed a subgroup analysis of samples by examining post‐surgical plasma collected during the first 4 weeks (days 0–28 post‐surgery) separately from those drawn at later timepoints (days 29–56 post‐surgery) (Figure 2).

During the 0–28 days post‐surgery period, 33 out of 46 (71.7%) patients had a cir‐nDNA amount higher than their pre‐surgery level (Figure 2A,D), with 23 out of 46 (50.0%) and 13 out of 46 (28.3%) being 2‐ and 3‐fold higher, respectively (Figure S1, Table S3A). Only 8 out of 46 (28.3%) cases showed post‐surgery cir‐nDNA levels comparable with those pre‐surgery (CV = 24%).

During the 28–56 days post‐surgery period, 19 out of 37 (51.4%) patients had cir‐nDNA levels higher than their pre‐surgery levels (Figure 2B,E), with 6 out of those 37 (28.7%) being two‐fold higher and 3 of 37 (8.11%) being three‐fold higher, respectively (Figure S1). Only 11 out of 37 (29.7%) showed comparable levels. The vast majority of cir‐nDNA concentrations increased post‐surgery, as compared to pre‐surgery values. In addition, data showed a trend toward higher elevations in the first 4 weeks post‐surgery (Figures 2A,D and S1). Results for the overall 0–56 day period, excluding the first sample when two samples were available for a given patient, are shown in Figure 2C,F.

**FIGURE 3:**
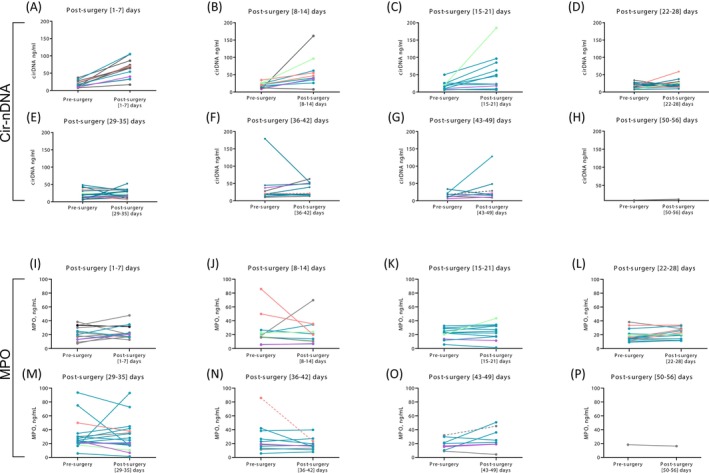
Comparative analysis of cir‐nDNA and MPO concentrations in pre‐surgery and every week in post‐surgery. The graphs representing per week the comparative analysis post‐surgery versus the pre‐surgery values. The analysis of Cir‐nDNA is showed for the first (A), second (B), third (C), fourth (D), fifth (E), sixth (F), seventh (G), eighth (H) week after surgery. The analysis of MPO showed for the first (I), second (J), third (K), fourth (L), fifth (M), sixth (N), seventh (O), eighth (P) week after surgery. Violet (category 1)—patients with no relapse or adverse event whose marker values return to healthy levels after 2 months; bleu (category 2)—patients with no relapse or adverse event whose marker values do not return to healthy levels after 2 months; green (category 3)—patients with relapse; rose (category 4)—patients with adverse events; gray (category 5)—patients without relapse or adverse events with less than 2 months follow up; gray dashed line—patients who got chemotherapy before sampling. Cir‐nDNA, circulating nuclear DNA; MPO, myeloperoxidase.

**FIGURE 4:**
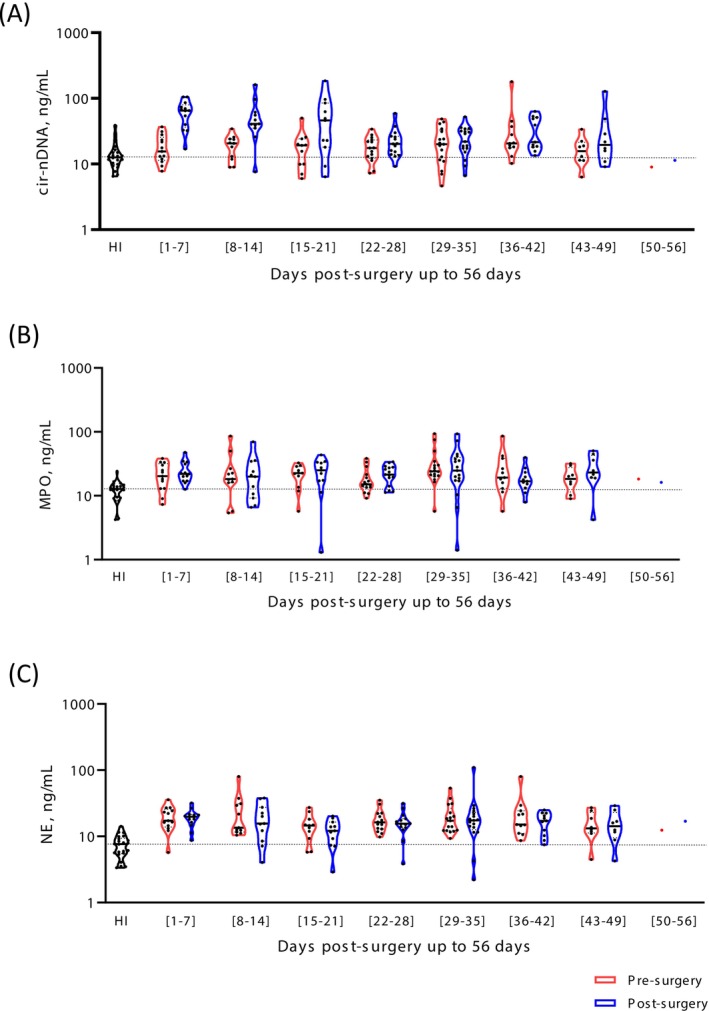
Comparative analysis of cir‐nDNA and MPO concentrations in pre‐surgery and every week in post‐surgery versus healthy individuals. The graphs representing the comparative analysis of circulating DNA (A), MPO (B), and NE (C) concentrations obtained post‐surgery are compared with the values obtained pre‐surgery and with the median obtained in the cohort of healthy individuals (HI). The bar plots are indicated in median with 95% CI. Cir‐nDNA, circulating nuclear DNA; MPO, myeloperoxidase; NE, neutrophil elastase.

In order to gain further insights into the fluctuations of cir‐nDNA levels, we considered the cir‐nDNA post‐operative values of plasma samples obtained within weekly periods up to 8 weeks post‐surgery. Whereas we observed a weak statistical difference between pre‐surgery and median cir‐nDNA levels in each of those 8 weeks post‐surgery, as compared to their matched pre‐operative values (Figure 3 and detailed in Table S5), stronger statistical differences were observed between control and post‐surgery samples during each week of the 8 week post‐surgery period studied (*p* <.05 to *p* <.0001, Table S5A). Detailed analysis of data obtained during each of the eight post‐surgery weeks is given in Suppl. material. Generally, the samples analyzed during the first week post‐surgery showed the strongest increase in cir‐nDNA levels, as compared to subsequent weeks. We observed a tendency for those levels to gradually decrease week on week up to the fourth week post‐surgery, while remaining elevated in the subsequent weeks up to the eighth week post‐surgery (100%, 90.9%, 81.8%, 73.3%, 58.8%, 72.7%, and 62.5% in the 1‐, 2‐, 3‐, 4‐, 5‐, 6‐, 7‐days post‐surgery week, respectively) (Figure S1 and Table S2). Increases in post‐surgery cir‐nDNA concentrations as compared to pre‐surgery appear to stabilize from the fifth to the eighth week post‐surgery (58.8%–72.7%). In addition, the median values obtained pre‐surgery or during the first 7 weeks post‐surgery are all statistically higher than the median of HIs (*p* <.05 to *p* <.0001, Table S5A). It is also significant that all median values obtained post‐surgery for each week are greater than the medians obtained pre‐surgery (Figure 4A). It should be noted that post‐surgery cir‐nDNA levels are statistically higher than pre‐surgery levels in only the first and second week post‐surgery (*p* <.0001, Table S5A).

When examining cir‐nDNA values of patients showing only a single post‐surgery sample (*N* = 47), we observed that 12 (25.5%) and 5 (10.6%) out of 47 patients had a lower value as compared to that patient's pre‐surgery value and the HI median value, respectively. Only 4 (8.5%) and 4 (8.5%) out of 47 patients showed a nearly unchanged value with respect to that patient's pre‐surgery value and the HI median value, respectively (Figure S2A). Conversely, 31 (66.0%) and 38 (80.8%) patients showed higher values as compared to matched patient's pre‐surgery value and the HI median value, respectively.

Twenty patients had two post‐surgery samples. We observed in those patients that 1 (5.0%) and 1 (5.0%) out of 20 patients showed lower or lower/similar values as compared to the matched patient's pre‐surgery value and the HI median value, respectively. Only 2 (10.0%) and 1 (5.0%) out of 20 patients showed an initial higher value followed by lower values as compared to the patient's pre‐surgery value and the HI median value, respectively. 17 (85.0%) and 18 (90.0%) out of 20 patients had higher or higher/similar values as compared to the patient's pre‐surgery value and HI median value, respectively (Figure S2B).

When examining all post‐surgery sample concentration values (N = 87), 0, 1, 0, 4, 4, 1, and 3 samples out of 13 (0%), 11 (9.1%), 11 (0%), 15 (26.%), 17 (23.5%), 11 (9.1%), and 8 (37.5%) samples, respectively, showed lower values than those determined pre‐surgery during the first, second, third, fourth, sixth, seventh week, respectively.

No strong trend emerges from the results when the plasmas originated from: (1) patients without relapse or adverse event whose marker values returned at 2 months post‐surgery to levels equivalent to those of healthy individuals; (2) patients without relapse or adverse events whose marker values at 2 months post‐surgery did not return to the levels of healthy controls; (3) patients experiencing a relapse or adverse event; and (4) patients without relapse or adverse event with less than 2 months of post‐surgery follow‐up (Figure 3A–H). Note, the plasmas of the two patients in our cohort who received chemotherapy before their post‐surgery blood sample was obtained showed a relative stability compared to their pre‐surgery values. Note that no correlation was observed between physically fit patients with rapid recovery and shorter sampling times and the concentration of total circulating DNA.

Using a chi‐squared test, we found no statistically significant relationship between MSS/MSI status and cirDNA variation patterns (χ^2^ = 0.93, df = 2, *p* = .63). The strength of association, as measured by Cramér's *V*, was weak (*V* = 0.13), further indicating that cirDNA variation post‐surgery is not meaningfully associated with microsatellite status in this cohort.

### 3.1 Follow‐up of the post‐surgery values of the NETs markers

The changes in MPO plasma concentrations as assessed at weekly intervals up to 8 weeks post‐surgery as a function of the pre‐surgery values is presented in Figure 3I–P. MPO concentration values in plasma samples taken during the first week post‐surgery increased in comparison to the baseline, with only two samples showing values lower than pre‐surgery. The MPO concentrations in samples drawn during the second week showed distinctly varied trends, and included both strong increases and decreases in value, as compared to pre‐surgery values. The MPO concentration values obtained during the third and fourth week (15–28 days post‐surgery) were relatively stable compared to pre‐surgery levels (Figure 3K,L). Post‐surgery MPO concentrations were mostly higher than those observed pre‐surgery, except for post‐surgery values obtained from the fifth to the eighth weeks, which remained relatively stable (Table S3B). Five plasmas showed very strong variations, both positive and negative (Figure 4B, Table S5B).

Altogether, all MPO values obtained pre‐surgery or during the first 8 weeks post‐surgery were statistically higher than the median in HIs (Figure 4B). Except during the sixth week post‐surgery, all post‐surgery values were found to be higher but not statistically different from the values obtained pre‐surgery. All NE concentrations determined pre‐surgery or during the first 8 weeks post‐surgery were statistically higher than the median of HIs (Table S3C). There was no significant difference between the concentrations determined for each week up to the eighth week post‐surgery (Figure 4C and Table S5C).

**FIGURE 5:**
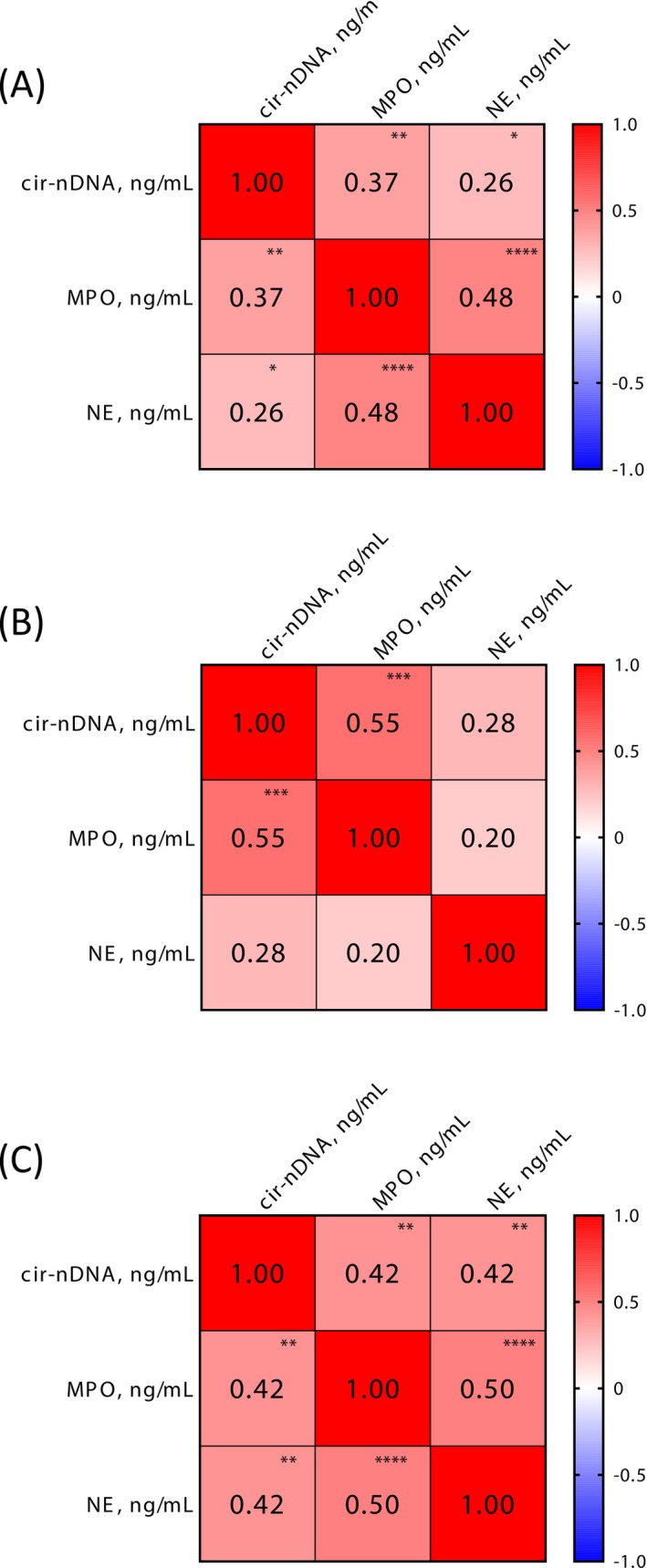
Correlation analysis of cir‐nDNA, MPO and NE concentrations in healthy individuals, pre‐surgery and post‐surgery for patients. Spearman correlation study between all MPO, NE and cir‐nDNA values. (A), pre‐surgery values (*n* = 74). Spearman *p*‐value associating MPO and NE, MPO and cir‐DNA and NE and cir‐nDNA are 1.60 × 10^−5^, 1.36 × 10^−3^ and 0.022; (B), 1–21 days of follow‐up values (*n* = 35). Spearman *p*‐value associating MPO and NE, MPO and cir‐DNA and NE and cir‐nDNA are 0.246, 5.71 × 10^−4^ and 0.110; (C), 22–56 days of follow‐up values (*n* = 52). Spearman *p*‐value associating MPO and NE, MPO and cir‐DNA and NE and cir‐nDNA are 1.76 × 10^−4^, 1.94 × 10^−3^, and 2.03 × 10^−3^, respectively. Cir‐nDNA, circulating nuclear DNA; MPO, myeloperoxidase.

Analysis of the association of NETs markers (MPO and NE) and total circulating DNA was performed using the Spearman correlation matrix (Figure 5). As previously observed, no association between cir‐nDNA and NETs markers was found in plasma from healthy individuals, whereas a significant association was found to exist between NE and MPO, which gives rise to their low‐level constitutive expression in HIs (Figure 5A). We observed a very strong association of the three markers (cir‐nDNA, MPO, and NE) during the first 3 weeks post‐surgery, and a slightly weaker association during the following 5 weeks (Figure 5B,C).

## 4 DISCUSSION

This is an ancillary study of the THRuST clinical study on stage III colon cancer. It focuses on the variation of cir‐nDNA levels within the first 8 weeks post‐surgery, in order to align with the timeframe within which the clinician must decide the type and duration of adjuvant therapy, guided by MRD detection as determined by cir‐nDNA analysis.

As expected, pre‐surgery cir‐nDNA concentrations were elevated by comparison to the HI median,^15^ with high levels being observed up to 2 weeks post‐surgery.^19^ Unexpectedly, we observed that post‐surgery cir‐nDNA concentrations generally do not decrease for up to 8 weeks post‐surgery, as compared to the median of HIs and to patients’ matched pre‐surgery levels. The differences between pre‐ and post‐surgery levels are greater in the first 2 weeks post‐surgery, and particularly so within the first week. In addition, cir‐nDNA levels may greatly increase in some patients, up to 18‐fold (Table S5). Only 18.9% of patient samples showed a significant decrease in cir‐nDNA levels in the 8 weeks post‐surgery, whereas it might have been expected that the trauma consequences of surgery (inflammation, cell death, et cetera) would not continue to impact cir‐nDNA concentration beyond the first month post‐surgery. Almost all cir‐nDNA concentrations as determined in the 8 weeks post‐surgery were higher than pre‐surgery levels, and did not decrease following surgery in cases where no relapse occurred. Only a few reports in the literature have addressed the determination of optimal timing of post‐operative blood sampling, and almost all of those are based on mutated cir‐nDNA (also conventionally but erroneously termed “ctDNA” in the literature, as explained above). Thus, there is currently no published work which considers pre‐ and post‐surgery cir‐nDNA (i.e., total circulating cell free nuclear DNA) where an accurate and validated method is employed during the first 2 months post‐surgery, and where the pre‐ and post‐surgery cir‐nDNA concentrations of each patient are compared. Our study is the first to reveal: (i), the high variation of post‐surgery cirDNA levels among stage III colon patients; and (ii), the elevated cirDNA level in most patients during the first 2 months of the post‐operative period. It should be noted that a small number of previous reports have shown data consistent with our observations.^20^ We and others^8^, ^21^ have previously inferred that the surgical procedure would enhance the release of cir‐nDNA and as a consequence we arbitrarily did not collect blood in the first 2 weeks post‐surgery.^8^, ^22^, ^23^

Two potential physiological effects linked to surgery may explain our observations: (i) “surgical trauma” denotes the physical damage inflicted on tissues during surgery, encompassing incisions, tissue manipulation, and organ removal, leading to local inflammation and tissue healing; and (ii) “surgical stress” refers to the combined physiological and psychological response engendered by the stressors associated with surgery, including the release of stress hormones and systemic changes in heart rate and immune function.^24^ While these two phenomena are related, they represent different aspects of the body's reaction to surgical procedures. Given that cir‐nDNA levels can remain elevated for up to 2 months post‐surgery, we assume that both phenomena might persist during the 2 months post‐operative period for stage III colon cancer patients.

Given that previous literature has linked an innate immune response involving an inflammatory process, namely the formation of NETs (Netosis), with cancer progression^25^, ^26^, ^27^ as well as with cir‐nDNA release,^10^ our study analyzed the presence of NETs markers (MPO and NE) within the same plasma samples in which cir‐nDNA content was assessed. NETs are scaffolds of DNA associated with various molecules, such as cytotoxic enzymes (MPO) or proteases (NE), which are released into the extracellular milieu.^28^ Their primary role is to control the spread of microbes during the first hours of infection, although dysregulated NETs production may lead to deleterious pathophysiological effects.^28^, ^29^ Our data clearly demonstrate that cir‐nDNA levels are strongly associated with NETs markers from the first to the eighth week post‐surgery. Neither of these observations has been reported previously, and as such they provide a new paradigm of both cir‐nDNA origin and the post‐surgery follow‐up of stage III colon cancer patients. This observation is consistent with our recent observations of stage II–III CRC and prostate cancer patients, in whom this association was revealed both in the immediate peri‐surgery period (1–72 h post‐surgery)^30^ and in follow‐ups performed up to 2 years post‐surgery, for the same patients as those considered in this study.^23^

Historically, investigations regarding the potential of using cir‐nDNA concentration as a standalone biomarker in oncology have tended to focus on a limited number of contexts: (i) evaluation of treatment response in metastatic disease; (ii) evaluation of the presence of recurrent cancer post‐surgery; (iii) the association of post‐surgery cancer recurrence with overall survival.^31^ Nevertheless, in patients with cancer it may be assumed that cir‐nDNA plasma concentration will range from just a few to several thousand ng/mL, which overlaps with the 1–20 ng/mL concentration range for healthy individuals.^15^, ^32^ Applying standardization and guidelines, Meddeb et al.^15^ compared the cir‐nDNA concentration of HIs (4 ng/mL, median; 0.4–22 ng/mL, range) and mCRC patients at diagnosis (13 ng/mL, median; 1–350 ng/mL, range). Because of its relatively low performance, total cir‐nDNA analysis was not at that point considered as a single biomarker. Indeed, numerous investigators have observed the progressive decrease in levels of circulating mutant DNA during the follow‐up period of tumor‐free patients.^3^

The significance of NETs as a principal source of cir‐nDNA in healthy and cancer individuals has recently been directly proven^11^ and is indirectly supported by cir‐nDNA fragmentome and methylome studies. In a milestone report,^33^ Shendure's team investigated the nucleosome occupancy as determined by fragmentomics and revealed that lymphoid or myeloid origins have the largest proportions consistent with hematopoietic cells as the dominant source of cir‐nDNA in healthy individuals, in contrast to patients with cancer. The work by Dor's team^34^ on cir‐nDNA methylome from HI also showed that white blood cells are among the main cir‐nDNA cells‐of‐origin (~85%), with granulocytes and erythrocyte progenitors being prominent amongst these. Recent reports have used methylome deconvolution to further characterize the neutrophil origin as one of the most important among a great variety of cells‐of‐origin,^34^ thus opening up a wide range of opportunities for diagnosis and for the interrogation of the process of tumor progression. While lower proportions of cir‐nDNA of neutrophil origin have been detected in patients with cancer than in HIs in normal conditions,^34^ of all the different sources of cir‐nDNA in patients with cancer, the neutrophil origin appears to be amongst the most important. A link has been made between inflammation, NETs and cir‐nDNA release in infectious diseases and sterile inflammatory diseases,^28^ such as cancer.^4^, ^10^ One possible explanation of this could be that NETs are produced from circulating neutrophils as well as from neutrophils within the tumor‐environment mass.

In addition, the contribution of NETs to post‐surgery cir‐nDNA concentrations appears to produce high inter‐individual variation, as was observed here among stage III colon cancer patients. There were variations in concentration of up to 11.3‐ and 18‐fold during the first and the second month, respectively.

The size of the patient cohort (*N* = 67) did not allow us to statistically evaluate whether cir‐nDNA or NETs markers are of value with regard to the prognosis of recurrence or adverse events, given the low proportion of relapse and adverse events in our cohort (14.9% and 6.8%, respectively). Another limitation of this study is the absence of an explanation of the high inter‐individual variability in NETs formation. To pursue that question, a much larger cohort, integrating detailed analysis of individual physiological and genetic/epigenetic factors, would be necessary.

The data suggest that it would be misleading to precisely define an optimal post‐surgery blood collection time for MRD detection. Given the high level of cir‐nDNA content and large inter‐individual variability, we estimate that the best time range for blood collection may be between the fourth and sixth week post‐surgery (Figure 4A).

Our observations appear to cast considerable doubt on the potential of post‐surgery MRD detection guided by cir‐mutDNA analysis, in particular in colorectal cancer (CRC) standard patient care. Thus, Tie et al.'s studies,^9^ for instance, proved that MRD detection through the use of cir‐nDNA analysis would greatly help the stratification of patients with regard to adjuvant therapy intensity. Given that these studies are based on the detection of cir‐nDNA bearing a mutation which shows the highest mutation allele frequency (MAF, proportion of mutant DNA among total DNA) as determined from blood samples by NGS, selection of the analysis of cir‐mutDNA bearing such a mutation may not always be reliable with respect to the level of WT cir‐nDNA, which may vary according to the individual patient. Given the low percentage of mutant cell clone which can sometimes occur within the tumor, the sensitivity of the detection is a critical issue with respect to MAF determination, due to the method's limit of detection (from 0.003% to 0.1%, using IntPlex qPCR and conventional NGS).^20^ Consequently, the selection of the mutation to be tracked post‐surgery, along with its monitoring from cir‐mutDNA based on MAF, are sub‐optimal. Furthermore, the doubtful reliability of using MAF from plasma is also a factor to be considered, for instance in the use of a MAF threshold for selecting patients for targeted therapy according to tumor mutation status.^21^, ^35^

Our observations point to a need to reassess the criteria for MRD blood collection time and for the use of MAF/VAF. Indeed, cir‐nDNA MAF should be considered a “false friend” marker when assessing the proportion of malignant cells within a tumor. With regard to these concerns, our data support the following recommendations: (i) for MRD detection, precise measurement of cir‐nDNA concentration must be carried out with appropriate methods; (ii) in the case of high cir‐nDNA concentrations (over a threshold, i.e., ~2‐fold control HI concentration), MPO and/or NE plasma concentration must be evaluated, and the most sensitive method available must be used to assess MAF; (iii) the absolute mutation quantification (concentration of cir‐mutDNA) must be used, rather than the relative MAF; and (iv) with regard to the selection of patients toward targeted therapy, the use of a MAF‐based threshold must not be used when determining tumor mutation status.

Altogether, this study reveals: (i) a high inter‐individual variability in post‐surgical cirDNA levels among stage III colon cancer patients; (ii) elevated cirDNA concentrations in most patients during the first 2 months following surgery; and (iii) an association between cir‐nDNA levels and NETs markers, suggesting that cir‐nDNA may also originate from NET degradation. These findings challenge previous assumptions in the field,^36^, ^37^ as most studies have speculated that cir‐nDNA levels would decline following curative treatment. Contrary to this expectation, our data show that the optimal window for blood collection is during the third and fourth postoperative weeks in most patients. Furthermore, our results support the use of absolute mutant cir‐nDNA concentrations—rather than MAF/VAF ratios—for more accurate MRD detection in CRC. Finally, our study underscores the potential role of NETs in cir‐nDNA release and suggests that monitoring NETs activity after surgery may be essential to mitigate their possible deleterious effects.

## AUTHOR CONTRIBUTIONS

**Andrei Kudriavtsev:** Writing – original draft; writing – review and editing; methodology; visualization; software; data curation. **Saidi Daoud:** Writing – original draft; data curation. **Catalina Isabel Cofre Muñoz:** Data curation. **Alexia Mirandola:** Data curation. **Ekaterina Pisareva:** Formal analysis. **Javier Gonzalo Ruiz:** Conceptualization. **Marco Macagno:** Data curation. **Nadia Saoudi Gonzalez:** Data curation. **Evelyne Crapez:** Validation. **Marc Ychou:** Supervision; investigation; conceptualization; funding acquisition. **Ramon Salazar Soler:** Supervision; conceptualization; investigation; funding acquisition. **Elisabetta Fenocchio:** Data curation; software. **Paula X. Fernandez Calotti:** Data curation. **Thibault Mazard:** Data curation; validation. **Cristina Santos Vivas:** Data curation; conceptualization. **Elena Elez:** Project administration; data curation; conceptualization; investigation; resources. **Federica Di Nicolantonio:** Project administration; conceptualization; supervision; writing – original draft; resources. **Alain R. Thierry:** Supervision; project administration; conceptualization; writing – original draft; review and editing.

## FUNDING INFORMATION

This work was supported by the European ERA‐NET grant on Translational Cancer Research (TRANSCAN‐2) “Minimally and non‐invasive methods for early detection and/or progression of cancer.” This work was partially supported by SIRIC Montpellier Cancer Grant INCa_Inserm_DGOS_12553, by MSD AVENIR [MSD‐THRuST grant], by the Société Française des Acides Nucléiques Circulants (SFAC) and by the RHU REVEAL, project reference ANR‐21‐RHUS‐0013. This work was supported in Spain by Fundación Científica Asociación Española Contra el Cancer (FC‐AECC)/Instituto de Salud Carlos III (ISCIII). This work was also supported in Italy by the Italian Ministry of Health.

## CONFLICT OF INTEREST STATEMENT

EE has held non‐remunerated roles with SEOM and ESMO and has served as a volunteer member of the ASCO Annual Meeting Scientific Program Committee and has received personal speaker honoraria from Organon and Novartis, advisory board honoraria from Amgen, Bayer, Hoffmann‐La Roche, Merck Serono, Sanofi, Pfizer, Pierre Fabre, MSD, and Servier, and travel and accommodation support from Amgen, Array Biopharma, Bristol‐Myers Squibb, Merck Serono, Pfizer, Roche, Sanofi, and Servier. FDN have received personal fees from Illumina for Webinars unrelated to the topic of this work. Other authors declare no potential conflicts of interest.

## ETHICS STATEMENT

Informed consent was obtained from all individuals and/or caregivers, and all clinical procedures and genetic testing, including data collection and report, were in accordance with the declaration of Helsinki and approved by the local ethical committees or followed other local guidelines: protocol 015‐FP018 by the Ethics Committee at the Hospital Universitari Bellvitge, protocol ID CE IRCCS n.233/2018 approved by the Ethical Committee of the Candiolo Cancer Institute FPO IRCCS, protocol 2019/34 approved by CPP Ouest II; protocol PR(AG)235/2018 approved by Vall d'Hebron Ethical Committee. The healthy individual control cohort was composed of samples from blood donors to the Etablissement Français du Sang (EFS, Montpellier, France). EFS blood samples are highly controlled and qualified. They are subjected to the same preanalytical conditions as for patients' blood samples before plasma analysis.

## Supporting information


**Data S1:** Supporting Information.

## Data Availability

Data sources and handling of the publicly available datasets used in this study are described in Table S1. The data that support the findings of this study are available from the corresponding author upon reasonable request.
