# Supplementary material for: Post‐surgery level of circulating DNA in stage III colon cancer patients: Impact on the reliability of minimal residual disease detection
Source: Int J Cancer. 2026 Feb 11;158(10):2771–83. doi: 10.1002/ijc.70370 (PMC12996737; doi:10.1002/ijc.70370)
Supplement: Supplementary file 1 — Data S1: Supporting Information. [file IJC-158-2771-s001.pdf]

Supplementary material

Post-surgery level of circulating DNA in stage III colon cancer patients: impact on the reliability of minimal residual disease detection

Andrei Kudriavtsev, Saidi Daoud, Catalina Isabel Cofre Muñoz, Alexia Mirandola, Ekaterina Pisareva, Javier Gonzalo Ruiz, Marco Macagno, Nadia Saoudi Gonzalez, Evelyne Crapez, Marc Ychou, Ramon Salazar Soler, Elisabetta Fenocchio, Paula X. Fernandez Calotti, Thibault Mazard, Cristina Santos Vivas, Elena Elez, Federica Di Nicolantonio and Alain R. Thierry.

Content of supplementary material

Supplementary Table 1 - Cir-nDNA, MPO and NE values detected in samples of patients.

Supplementary Table 2 - A – Overhaul cir-nDNA concentration before and after surgery over an 8-week period; B - analysis the x-folding factor of circulating nuclear DNA for each week of follow-up

Supplementary Table 3 - Comparative characteristics of circulating nuclear DNA (A), myeloperoxidase (B) and neutrophils elastase (C) values for patient groups in different periods of follow-up

Supplementary Table 4 - Determination of cir-nDNA, MPO and NE healthy individual median concentration values and positivity thresholds

Supplementary Table 5 - Statistical comparison of circulating nuclear DNA (A), myeloperoxidase (B) and neutrophils elastase (C) values before and after surgery for patient up to 8 weeks of follow-up

Supplementary Figure 1 - Proportion of patients showing greater post-surgery values as compared to healthy individuals (HI) median and pre-surgery values in respect to the concentration of circulating nuclear DNA (A), myeloperoxidase (B) and neutrophils elastase (C).

Supplementary Figure 2 - The swimmer plot illustrating the timeline of blood sample collection for patients with 1 post-surgery sample (A) and for patients with 2 post-surgery samples (B).

Supplementary information - Detailled analysis of Figure 3 A-H

Supplementary Table 1

ID patient	Blood collection time (Day)	cir-nDNA, ng/mL	MPO, ng/mL	NE, ng/mL
01-002	0	15.50	16.9	16.5
	4	64.60	22.3	19.8
	27	29.90	21.4	15.1
01-003	0	7.28	28.77	12.95
	27	9.20	32.84	13.53
01-006	0	9.90	32.65	27.35
	21	49.34	34.32	20.28
01-011	0	7.80	30.1	12.2
	4	16.90	33.8	8.7
	34	15.20	35.4	17.4
01-021	0	10.90	33.2	23.2
	4	73.60	47.8	26.7
01-023	0	21.70	38.4	22.2
	4	68.30	20.0	20.2
	24	23.90	28.9	17.4
01-029	0	23.20	33.55	16.23
	4	69.90	31.43	18.26
	28	25.80	33.94	18.45
01-033	-3	18.24	31.82	24.43
	45	28.83	45.12	29.03
01-035	0	48.43	27.10	13.97
	35	31.04	41.68	13.67
01-036	0	13.20	25.04	35.78
	4	32.88	17.05	31.98
01-048	0	19.13	21.35	12.30
	31	30.70	28.13	16.43
01-050	-1	9.30	12.9	5.7
	3	40.00	20.4	18.1
01-053	0	179.27	42.00	15.10
	42	53.31	15.29	7.47
01-054	0	18.93	20.172	18.68
	45	15.94	21.505	16.85
01-070	0	6.33	16.567	12.74
	45	10.89	19.310	8.82
01-077	0	34.00	13.64	19.87
	28	15.34	11.68	3.84
01-081	0	22.22	10.17	11.03
	49	128.02	36.06	15.95
01-087	0	11.42	15.28	13.71
	49	21.74	18.92	11.62
01-092	0	20.90	26.6	21.7
	9	40.50	21.0	20.0
	37	18.10	19.7	18.8
01-093	0	36.80	23.4	27.0
	5	86.00	15.9	11.3
01-101	0	11.34	14.71	13.24
	28	14.03	27.88	15.42
01-105	0	44.77	38.276	21.18
	38	48.77	39.687	20.53
01-108	0	11.86	16.41	10.44
	8	7.62	10.68	4.05
01-123	-3	10.23	11.46	8.62
	36	13.47	13.21	8.69
01-124	0	21.90	9.15	11.05
	23	16.07	11.96	8.48
01-125	0	16.33	23.94	12.40
	34	6.64	6.54	2.21
01-130	0	31.10	20.2	17.2
	4	105.20	34.7	14.7
	30	34.80	21.1	20.0
01-136	0	18.30	16.0	29.5
	13	26.00	13.9	38.0
	37	19.50	17.8	22.7
01-139	0	27.90	18.3	24.3
	4	65.00	12.6	21.1
	41	63.10	16.8	25.0
01-141	-15	25.40	21.4	30.6
	28	13.10	19.5	26.5
01-144	0	24.00	17.9	31.2
	8	40.90	9.2	22.5
	35	22.00	10.4	26.4
01-150	0	23.70	21.87	11.65
	10	61.60	34.66	7.16
	30	17.40	18.73	11.52
01-157	0	17.60	13.0	14.5
	4	54.20	22.0	21.6
	38	15.60	11.1	17.1
01-168	0	7.00	5.7	9.3
	19	6.40	1.3	7.1
	34	11.40	1.4	11.6

Supplementary Table 1 - continued

ID patient	Blood collection time (Day)	cir-nDNA. ng/mL	MPO. ng/mL	NE. ng/mL
01-169	0	9.00	18.3	12.4
	12	161.90	69.7	37.1
	50	11.40	16.2	16.9
01-171	0	11.90	7.3	13.2
	4	104.90	22.9	20.9
01-172	0	13.10	9.0	27.2
	3	32.20	17.0	16.4
	48	9.10	4.2	12.5
01-178	0	13.10	5.7	11.1
	9	35.20	6.6	12.2
	37	18.60	7.9	12.4
01-183	0	17.60	15.1	22.4
	28	18.90	19.1	15.6
02-001	33	35.74	72.602	24.14
	-23	41.00	93.527	30.47
02-002	-28	43.67	75.01	53.46
	31	9.38	17.03	14.24
02-004	15	62.39	17.116	7.36
	-1	15.58	11.865	5.78
02-007	-4	8.92	5.39	12.53
	13	38.72	7.00	8.51
02-018	21	185.06	43.58	11.55
	-4	19.45	20.33	14.54
02-022	21	96.47	27.39	14.12
	-2	49.99	28.78	14.80
02-028	0	4.64	16.44	19.15
	29	52.42	92.99	110.09
02-035	-1	12.26	13.51	17.49
	28	20.30	25.98	31.54
03-001	-28	20.61	85.87	79.91
	13	47.03	19.91	15.36
	41	21.53	23.28	16.61
03-002	-26	25.70	22.54	13.53
	8	96.36	23.90	15.58
	16	22.86	22.33	12.11
03-004	-22	11.55	24.82	20.04
	32	31.66	34.23	21.84
03-005	-20	19.21	22.84	14.92
	15	46.14	25.04	16.45
	36	39.17	27.14	22.49
03-006	-16	34.39	49.86	37.53
	13	55.45	35.49	27.50
	33	34.40	37.74	29.29
03-008	-1	27.85	14.37	9.80
	26	20.50	14.10	12.53
03-009	-27	33.73	29.82	11.93
	43	17.30	24.77	4.25
03-010	-51	20.02	29.82	11.93
	17	22.98	33.32	9.24
	32	29.00	24.77	4.25
03-011	-6	20.80	34.59	13.98
	29	19.00	44.80	18.28
03-012	-8	14.01	10.81	15.26
	28	37.45	11.28	16.69
04-003	-5	7.76	19.10	11.76
	28	26.54	20.83	13.97
04-004	0	13.05	16.22	15.09
	27	13.56	27.36	15.55
04-009	-1	24.24	24.96	23.59
	19	85.01	32.48	13.94
04-012	-9	11.02	20.61	18.58
	35	17.39	19.86	15.48
04-014	-1	12.84	24.00	22.73
	30	18.82	17.31	17.79
04-016	-1	37.21	19.25	10.62
	40	51.00	16.13	10.27
04-019	-1	9.98	13.64	5.86
	16	17.99	11.32	2.90
04-021	0	17.49	11.12	35.27
	28	58.96	23.47	20.79
04-032	-15	6.00	22.20	18.11
	21	9.23	17.44	18.91
04-037	-1	11.31	21.10	4.48
	44	48.63	50.65	25.03

Suppl. Table 1: Cir-nDNA, MPO and NE values detected in samples of patients. Cir-nDNA- circulating nuclear DNA; MPO – myeloperoxidase, NE - neutrophils elastase, 0 – surgery day.

Supplementary Table 2

A

Days	n patients	Post-surgery, cir-nDNA (ng/ml)						
		min	max	Ratio max/min	Median	SD	Mean	CV, %
1-7	13	16.9	105.2	6.2	65.0	27.1	62.6	43.4%
8-14	11	7.6	161.9	21.3	40.9	41.7	55.6	75.1%
15-21	11	6.4	185.1	28.9	46.1	52.5	54.9	95.7%
22-28	15	9.2	59.0	6.4	20.3	12.4	22.9	54.3%
29-35	17	6.6	52.4	7.9	22.0	11.8	24.5	48.2%
36-42	11	13.5	63.1	4.7	21.5	18.3	32.9	55.6%
43-49	8	9.1	128.0	14.1	19.5	39.6	35.1	113.0%

B

Days	n patients	x-fold, min	x-fold, max	x-fold, Median	x-fold, Mean	x-fold, SD	x-fold, CV, %
1-7	13	2.17	8.82	3.08	3.7	1.97	52.77%
8-14	11	0.64	17.99	2.28	3.7	4.85	130.12%
15-21	11	0.89	9.52	1.93	3.0	2.55	85.90%
22-28	15	0.45	3.42	1.11	1.5	0.96	64.38%
29-35	17	0.21	11.30	1.12	1.8	2.52	140.55%
36-42	11	0.30	2.26	1.09	1.2	0.55	43.98%
43-49	8	0.51	5.76	1.65	2.2	1.88	86.84%

Suppl. Table 2: A – Overhaul cir-nDNA concentration before and after surgery over an 8-week period; B - analysis the x-folding factor of circulating nuclear DNA for each week of follow-up. SD – standard deviation, CV - coefficient of variation.

Supplementary Table 3

A

Days post-surgery	Nb patients	Pre-surgery median cir-nDNA, ng/mL	Pre-surgery, CV, %	Post-surgery median cir-nDNA, ng/mL	Post-surgery, CV, %	post > median HI, nb	post > median HI, %	post > pre, nb	post > pre, %	post > pre 2x fold, nb	post > pre 2x fold, %	post > pre 3x fold, nb	post > pre 3x fold, nb
1-7	13	15.5	49.0%	65.0	43.4%	13	100%	13	100%	13	100%	8	62%
8-14	11	20.6	41.2%	40.9	75.1%	10	91%	10	91%	6	55%	3	27%
15-21	11	19.2	65.5%	46.1	95.7%	9	82%	8	73%	5	45%	4	36%
22-28	15	17.5	42.2%	20.3	54.3%	14	93%	6	40%	3	20%	2	13%
29-35	17	20.0	60.2%	22.0	48.2%	14	82%	8	47%	2	12%	1	6%
36-42	11	20.6	129.6%	21.5	55.6%	11	100%	5	45%	2	18%	0	0%
43-49	8	15.7	50.2%	19.5	113.0%	6	75%	5	63%	2	25%	2	25%
50-56	1	9.0		11.4		0	0%	1	100%	0	0%	0	0%

B

Days post-surgery	Nb patients	Pre-surgery median MPO, ng/mL	Post-surgery median MPO, ng/mL	post > median HI, nb	post > median HI, %	post > pre, nb	post > pre, %	post > pre 2x fold, nb	post > pre 2x fold, %	post > pre 3x fold, nb	post > pre 3x fold, nb
1-7	13	20.2	22.0	12	92%	8	62%	1	8%	1	8%
8-14	11	18.3	19.9	7	64%	4	36%	1	9%	1	9%
15-21	11	22.5	25.0	9	82%	4	36%	1	9%	0	0%
22-28	15	15.1	21.4	12	80%	8	53%	1	7%	0	0%
29-35	17	24.0	24.8	14	82%	6	35%	1	6%	1	6%
36-42	11	19.3	16.8	9	82%	4	36%	0	0%	0	0%
43-49	8	18.4	24.2	7	88%	5	63%	2	25%	1	13%
50-56	1	18.3	16.2	1	100%	0	0%	0	0%	0	0%

C

Days post-surgery	Nb patients	Pre-surgery median NE, ng/mL	Post-surgery median NE, ng/mL	post > median HI, nb	post > median HI, %	post > pre, nb	post > pre, %	2x fold, nb	post > pre 2x fold, %	post > pre 3x fold, nb	post > pre 3x fold, nb
1-7	13	17.2	19.8	13	100%	6	46%	1	8%	1	8%
8-14	11	13.5	15.6	9	82%	4	36%	1	9%	0	0%
15-21	11	14.5	12.1	8	73%	2	18%	0	0%	0	0%
22-28	15	16.2	15.6	14	93%	5	33%	0	0%	0	0%
29-35	17	17.2	17.4	15	88%	6	35%	1	6%	1	6%
36-42	11	15.1	17.1	10	91%	3	27%	0	0%	0	0%
43-49	8	14.9	14.2	8	100%	3	38%	1	13%	1	13%
50-56	1	12.4	16.9	1	100%	1	100%	0	0%	0	0%

Suppl. Table 3: Comparative characteristics of circulating nuclear DNA (A), myeloperoxidase (B) and neutrophils elastase (C) values for patient groups in different periods of follow-up. Cir-nDNA: circulating nuclear DNA; MPO – myeloperoxidase, NE - neutrophils elastase. Note, CV of the cirDNA concentration value obtained from one plasma sample is (24%) (Meddeb et al, Sci Rep, 2019)

Supplementary Table 4

ID	cir-nDNA, ng/mL	MPO, ng/mL	NE, ng/mL
EFS 1520	10.3	8.34	8.62
EFS 4969	8.9	12.41	7.59
EFS 262	13.3	14.72	8.01
EFS 3841	13.2	13.54	5.65
EFS 5900	22.1	12.02	3.48
EFS -385	6.9	16.63	11.51
EFS 6161	15.0	13.88	8.46
EFS 4567	18.5	14.03	7.88
EFS 1061	6.5	12.41	11.37
EFS 6153	12.4	**24.18**	7.69
EFS 7469	9.3	14.13	5.57
EFS 1109	11.7	8.29	3.35
EFS 5935	12.9	9.23	9.20
EFS 7739	11.5	9.52	6.10
EFS 5927	**27.9**	15.50	**14.16**
EFS 1539	11.7	9.08	5.81
EFS 1555	14.9	12.36	5.36
EFS 6196	13.5	13.34	5.97
EFS 2531	9.9	4.23	3.53
EFS 9005	16.5	13.15	9.94
EFS 6188	**38.8**	15.70	10.08
EFS 0624	7.7	4.42	4.06

Median	*12.64*	*12.78*	*7.64*
SD	*7.22*	*4.19*	*2.81*
Mean	14.25	12.32	7.43
CV%	*50.67*	*34.00*	*37.77*

Suppl. table.4: Determination of cir-nDNA, MPO and NE healthy individual median concentration values and positivity thresholds. Bold values indicate values above the two-fold over the median value threshold suggesting some significant variation of the marker value in these healthy individuals.

Supplementary Table 5

A

**Test de Mann-Whitney**	DATA Peri-THRuST VS HI		Pre VS Post-surgery	
	p-value	p-value summary	p-value	p-value summary
Pre-surgery associated with "Post-surgery [1-7] days"	0.1509	ns	< 0.0001	****
Post-surgery [1-7] days	< 0.0001	****		
Pre-surgery associated with "Post-surgery [15-21] days"	0.0807	ns	0.0008	***
Post-surgery [8-14] days	< 0.0001	****		
Pre-surgery associated with "Post-surgery [15-21] days"	0.2855	ns	0.0759	ns
Post-surgery [15-21] days	0.0042	**		
Pre-surgery associated with "Post-surgery [22-28] days"	0.0847	ns	0.2671	ns
Post-surgery [22-28] days	0.0023	**		
Pre-surgery associated with "Post-surgery [29-35] days"	0.0621	ns	0.6098	ns
Post-surgery [29-35] days	0.0023	**		
Pre-surgery associated with "Post-surgery [36-42] days"	0.003	**	0.4779	ns
Post-surgery [36-42] days	< 0.0001	****		
Pre-surgery associated with "Post-surgery [43-49] days"	0.4692	ns	0.5054	ns
Post-surgery [43-49] days	0.0491	*		
Pre-surgery associated with "Post-surgery [50-56] days"	N=1, pas de statistiques possibles			
Post-surgery [50-56] days				

B

**Test de Mann-Whitney**	DATA Peri-THRuST VS HI		Pre VS Post-surgery	
	p-value	p-value summary	p-value	p-value summary
Pre-surgery associated with "Post-surgery [1-7] days"	0.006	**	0.5446	ns
Post-surgery [1-7] days	< 0.0001	****		
Pre-surgery associated with "Post-surgery [15-21] days"	0.0042	**	0.8977	ns
Post-surgery [8-14] days	0.1409	ns		
Pre-surgery associated with "Post-surgery [15-21] days"	0.0037	**	0.519	ns
Post-surgery [15-21] days	0.0013	**		
Pre-surgery associated with "Post-surgery [22-28] days"	0.0102	*	0.1703	ns
Post-surgery [22-28] days	0.0004	***		
Pre-surgery associated with "Post-surgery [29-35] days"	< 0.0001	****	0.8119	ns
Post-surgery [29-35] days	0.0002	***		
Pre-surgery associated with "Post-surgery [36-42] days"	0.0063	**	0.519	ns
Post-surgery [36-42] days	0.0104	*		
Pre-surgery associated with "Post-surgery [43-49] days"	0.0264	*	0.2345	ns
Post-surgery [43-49] days	0.002	**		
Pre-surgery associated with "Post-surgery [50-56] days"	N=1, pas de statistiques possibles			
Post-surgery [50-56] days				

Supplementary Table 5 - continued

C

**Mann-Whitney test**	DATA Peri-THRuST VS HI		Pre VS Post-surgery	
	p-value	p-value summary	p-value	p-value summary
Pre-surgery associated with "Post-surgery [1-7] days"	< 0.0001	****	0.9197	ns
Post-surgery [1-7] days	< 0.0001	****		
Pre-surgery associated with "Post-surgery [15-21] days"	< 0.0001	****	0.6994	ns
Post-surgery [8-14] days	0.0011	**		
Pre-surgery associated with "Post-surgery [15-21] days"	0.0007	***	0.4779	ns
Post-surgery [15-21] days	0.0107	*		
Pre-surgery associated with "Post-surgery [22-28] days"	< 0.0001	****	0.8063	ns
Post-surgery [22-28] days	< 0.0001	****		
Pre-surgery associated with "Post-surgery [29-35] days"	< 0.0001	****	0.8384	ns
Post-surgery [29-35] days	< 0.0001	****		
Pre-surgery associated with "Post-surgery [36-42] days"	< 0.0001	****	0.6994	ns
Post-surgery [36-42] days	< 0.0001	****		
Pre-surgery associated with "Post-surgery [43-49] days"	0.0017	**	0.9591	ns
Post-surgery [43-49] days	0.0025	**		
Pre-surgery associated with "Post-surgery [50-56] days"	N=1, no statistics available			
Post-surgery [50-56] days				

Suppl. Table 5: Statistical comparison of circulating nuclear DNA (A), myeloperoxidase (B) and neutrophils elastase (C) values before and after surgery for patient up to 8 weeks of follow-up. Mann Whitney test. Cir-nDNA: circulating nuclear DNA; MPO – myeloperoxidase, NE - neutrophils elastase.

Supplementary Figure 1

(A)

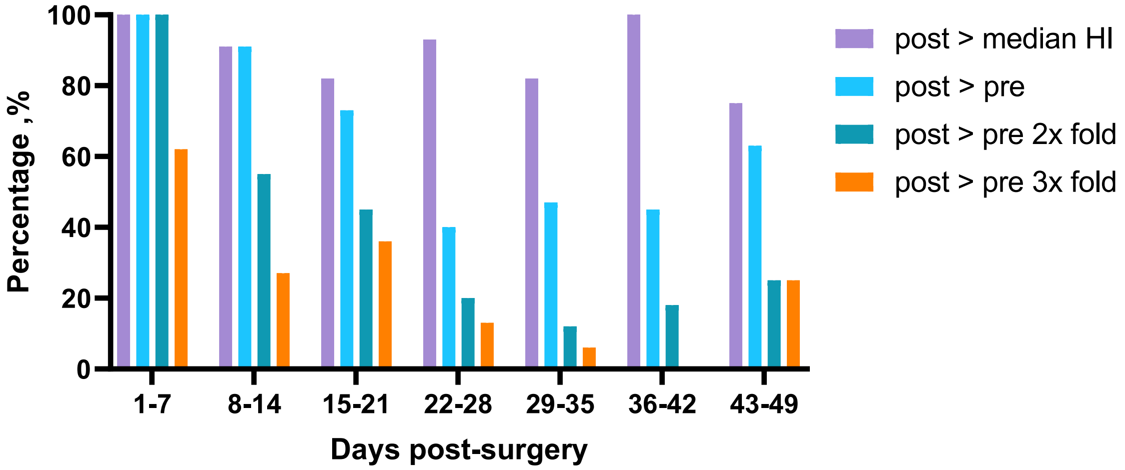

(B)

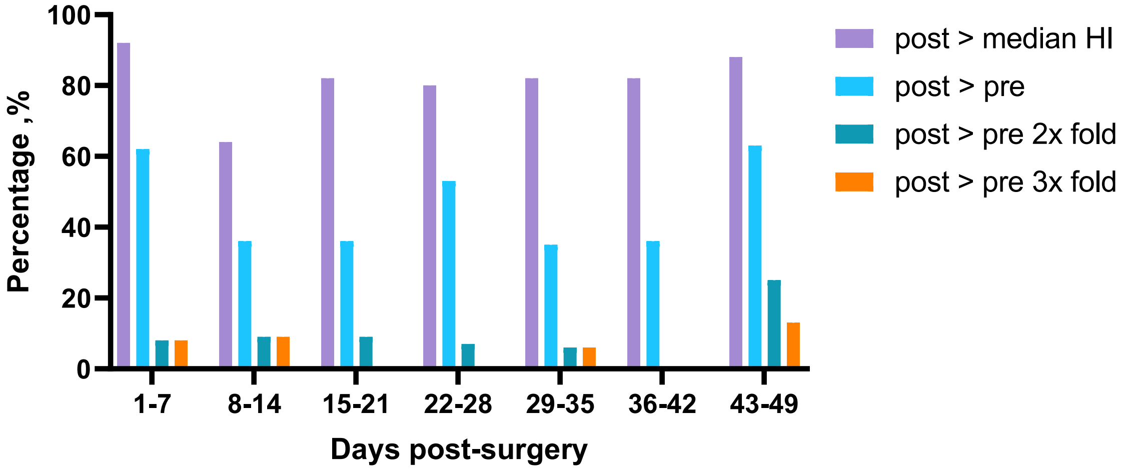

(C)

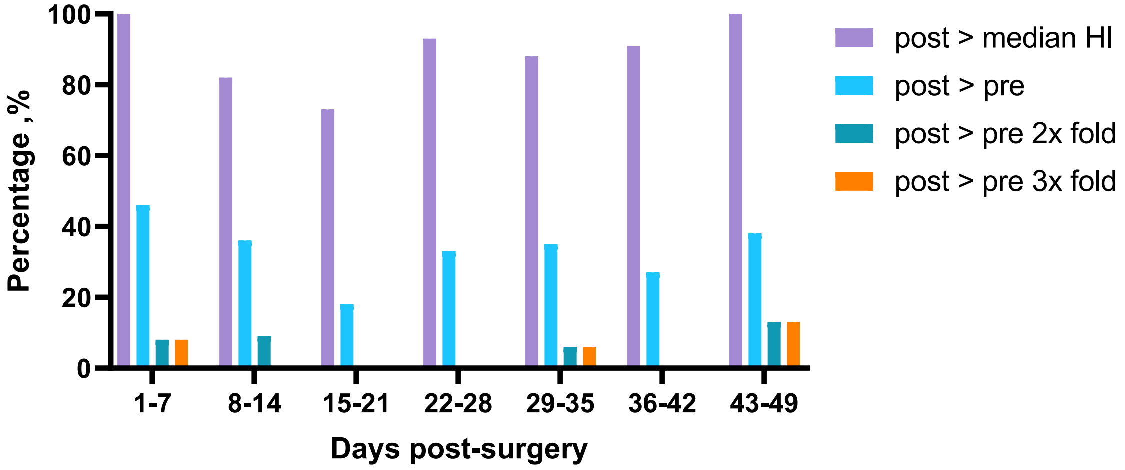

Suppl. figure 1: Proportion of patients showing greater post-surgery values as compared to healthy individuals (HI) median and pre-surgery values in respect to the concentration of circulating nuclear DNA (A), myeloperoxidase (B) and neutrophils elastase (C).

Supplementary figure 2

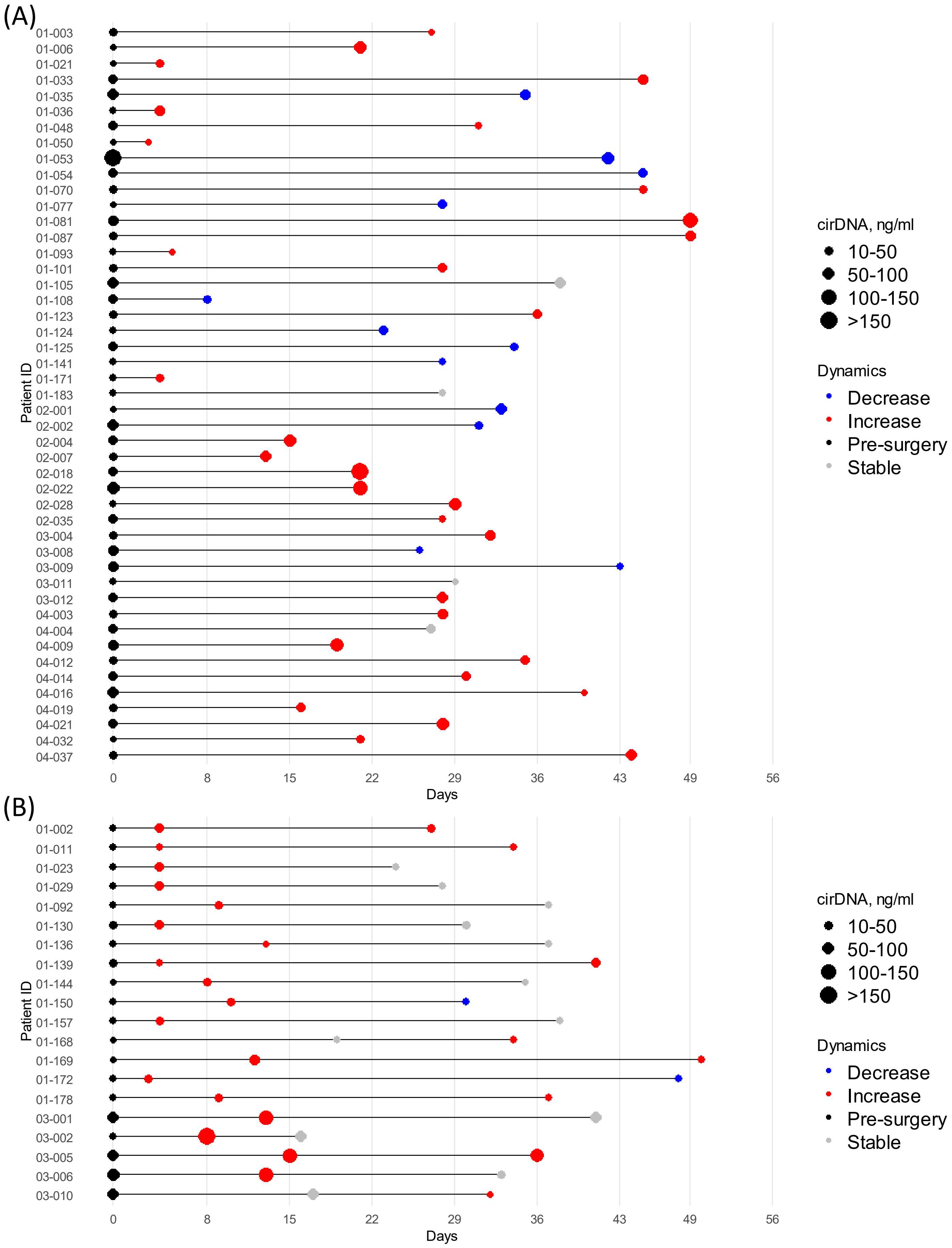

Suppl. figure 2: The swimmer plot illustrating the timeline of blood sample collection for patients with 1 post-surgery sample (A) and for patients with 2 post-surgery samples (B).

Detailled analysis of Figure 3 A-H

During the first week post-surgery (Figure 3A), all 13 patients displayed at least a twofold increase in cirDNA levels, as compared to their matched pre-surgery value. In the subsequent [8-14] days post-surgery period (Figure 3B), 91% of the plasma samples showed elevated cirDNA levels. Among these, 27% experienced a twofold increase, while 55% demonstrated a threefold post-surgery increase in cirDNA concentration (Fig 3B). During the third week post-surgery ([15-21] days, Figure 3C), 73% of the samples displayed elevated cirDNA levels, as compared to matched pre-surgery samples. Among these, 18% and 46% exhibited a two- and three-fold increase in cirDNA concentrations. Among the samples analyzed during the [22-28] days post-surgery period (Figure 3D), 47% showed higher cirDNA levels post-surgery, with 20% showing a two-fold increase, 20% demonstrating a three-fold increase, and 27% maintaining stable cirDNA levels. Analysis from the fifth post-surgery week ([29-35] days) showed 47% of samples with an increase in post-operative cirDNA levels, with 24% maintaining stable cirDNA concentrations, and 24% and 12% showing a two- and a three-fold increase in cirDNA levels (Figure 3E). 45% of samples tested within the sixth post-operative week ([36-42] days, Figure 3F) showed elevated cirDNA levels, with 18% and x% showing a two- and three-fold increase in cirDNA concentrations. Within the [43-49] days post-surgery timeframe (post-surgery seventh week, Figure 3G), 62% of patients had an increase in cirDNA concentrations, and 25% and x% of the samples showed a two- and three-fold increase. Lastly, the only plasma tested during the eighth week post-surgery ([50-56] days post-surgery Figure 3H) showed an x-fold increase in cirDNA level, as compared to the preoperative baseline value.
